# Effect of COVID-19 lockdown on Moroccan patients with type 1 and type 2 diabetes

**DOI:** 10.1186/s42269-022-00827-8

**Published:** 2022-05-16

**Authors:** Rochdi Kaddar, Chayma Tarik, Maryam Atmani, Ikrame Enakhil, Nada Fakhri, Mohamed Khalis, Abdellah Lotfy, Nadia El Kadmiri

**Affiliations:** 1 International School of Public Health, Mohammed VI University of Health Sciences, Casablanca, Morocco; 2 Regional Direction of Health and Social Protection, Souss Massa Region, Agadir City, Morocco; 3 High Institute of Nursing Professions and Health Techniques of Agadir, Agadir City, Morocco; 4 Molecular Engineering, Valorization and Environment Team, Polydisciplinary Faculty of Taroudant, IBN ZOHR University, B.P. 271, 83 000 Taroudannt City, Morocco

**Keywords:** COVID-19 lockdown, Diabetes, HbA1c, Moroccan patients

## Abstract

**Background:**

The implementation of coronavirus disease of 2019 (COVID-19) lockdown has affected the daily practices of subjects with chronic diseases such as diabetes and caused negative impact on their lifestyle and habits such as physical activity, dietary habits and accessibility to medications. Diabetic people are considered the most vulnerable groups to COVID-19, and the lockdown measure has disturbed the diabetes self-management. In our study, we aimed to assess, for the first time at the regional level (Souss Massa Region), the COVID-19 lockdown impact on HbA1c levels in patients with type 1 diabetes (T1D) and type 2 diabetes (T2D). We carried out a cross-sectional quantitative analysis at the health center of the industrial district in Agadir City.

**Results:**

We found a significant improvement in post-lockdown mean ± SD HbA1c in 150 subjects suffering from T1D and T2D; *p* = 0.005). Our analysis revealed a significant association of HbA1c deviation with educational level and medical coverage (*p* = 0.01). No significant association was detected between HbA1c deviation and age, gender, weight, height, current BMI status, fasting blood sugar, family history, urban or rural areas, marital status, professional activity, socioeconomic income, type of diabetes, dietary, comorbidities, diabetic complications, housing, adherence to the dietary recommendations, physical activity, medical appointments, stopping medication, self-monitoring, fasting and anxiety about getting COVID-19.

**Conclusions:**

COVID-19 lockdown had no deleterious effect on HbA1c levels in Moroccan patients with T1D and T2D.

## Background

In December 2019, a new beta coronavirus of severe acute respiratory syndrome coronavirus 2 (SARS-CoV-2) had emerged in Wuhan City, China. The transmission rate of the infection, termed COVID-19 by World Health Organization (WHO), was extremely high and it spread rapidly worldwide leading to a global pandemic. On March 11, 2020, WHO had declared COVID-19 as a pandemic. Globally, 216,229,741 coronavirus-confirmed cases and 4,496,681 deaths have been reported in August 2021 according to WHO declarations. In Morocco, COVID-19 led to 853,373 cases and claimed 12,437 lives in August 2021, according to WHO Coronavirus (COVID-19) Dashboard (WHO 2021). In order to mitigate and slow the spread of the disease, public health authorities applied exceptional measures in many parts of the world; social distancing and lockdowns. On March 20, 2020, Morocco established a highly strict lockdown until June 10, 2020, and citizens were advised to remain indoors except for limited necessities such as emergency medical help or alimentary shopping (Cucinotta and Vanelli 2020; Moroccan-Ministry-of-Health; WHO). The implementation of COVID-19 lockdown has affected the daily practices of subjects with chronic diseases such as diabetes and caused negative impact on their lifestyle and habits such as physical activity, dietary habits and accessibility to medications.

Diabetes is defined as a chronic metabolic disorder characterized by elevated blood glucose, which includes T1D and T2D. The chronic hyperglycemia is associated with several complications and pathological implications. The major indicator for diabetes assessment is glycated hemoglobin or HbA1c. HbA1c values signify the levels of blood sugar over the previous 2–3 months. Diabetic complications are associated with higher HBA1c (Orban and Ichai 2008). In 2019, WHO estimated 463 million diabetics aged 19 to 79 worldwide (9% for women–9.6% for men) and 1.5 million deaths were directly caused by diabetes. In Morocco, the prevalence in the adult population is 12.4%. Diabetes kills over 12,000 people a year (WHO 2021).

## Objectives

Diabetic people are considered the most vulnerable groups to COVID-19, and the lockdown measure has disturbed the diabetes self-management. Within this context, we aimed to assess, for the first time at the regional level (Souss Massa Region), the COVID-19 lockdown impact on HbA1c levels in patients with T1D and T2D.

## Methods

### Study design

Our cross-sectional quantitative analysis was carried out from April 9, 2021, to June 6, 2021, at the health center of the industrial district in Agadir City. According to eligibility criteria, 150 patients were included in this study.

### Data collection

Anonymized data were generated from a structured questionnaire created in Sphinx software by taking into consideration the education level of the interviewed patients.

The validated questionnaire consisted of four parts:

- Part 1: socio-demographic characteristics including twelve questions (sex, place of residence, age, weight before and after lockdown, height, BMI, level of education, family status, occupation, medical and socioeconomic level according to annual income).
- Part 2: disease characteristics including seventeen questions (type and duration of diabetes, therapeutic regimen including dosage and information about therapeutic agents, HbA1c and fasting blood sugar values before and after lockdown, comorbidity, complications and the frequency of monitoring).
- Part 3: patients’ attitudes comprising twenty questions (housing, dietary; based on 4 items related to compliance with clinician's dietary recommendations and the degree of adherence to dietary guidelines (Gray and Threlkeld 2000; American Diabetes Association 2019), physical activity ≥ three times 30 min per week: based on 4 items and estimated scale of 10, reducing or stopping medication, self-monitoring, anxiety about getting COVID-19, and fasting the holy month of Ramadan).
- Part 4: COVID-19 infection comprising three questions (COVID-19 infection, vaccination and number of doses received).

### Participants: inclusion criteria

- Confirmed T1D or T2D based on medical records; fasting blood sugar > 126 mg/dl; HbA1C > 7%
- Aged ≥ 18 years with a ≥ 1 year T1D and T2D diagnosis
- Recruited and monitored at the Health Center of the industrial district in Agadir City.

### Exclusion criteria

- Incomplete medical records
- No HBA1c records in the last 3 months
- Change of antidiabetic treatment at least 6 months before the lockdown and during the lockdown
- Pregnancy
- Active oncological illness (confirmed malignancy; under immunotherapy or chemotherapy)
- Comorbidity with psychiatric, neurological disorders and non-communicable diseases

### Data analysis

Anonymized data were divided according to two different periods: pre-lockdown and post-lockdown. Statistical analysis was performed using IBM SPSS Statistics 23 software. The degree of association was assessed using odds ratio (OR) (95% confidence intervals). A *p* value ≤ 0.05 was considered statistically significant.

#### Determined variables

- Dependent variables: glycemic balance (diabetes balance)
- Independent variables: diabetes, COVID-19, confinement, socio-demographic factors and attitudes.
- HbA1c deviation = HbA1c post-COVID-19-lockdown − HbA1c pre-COVID-19-lockdown [If the value is less than or equal to 0: stable/improved; otherwise greater than 0: aggravated]

### Ethical consideration

- The study was conducted in accordance with Helsinki Declaration. Authorization was obtained by Souss Massa Regional Direction of Health before starting the study while respecting the confidentiality of the data and the respect of anonymity in the processing of the data. All participants gave verbal informed consent to answer the anonymous questionnaire.

## Results

Overall, 150 participants (T1D: 46.7%, T2D: 53.3%) completed the survey questionnaire, 52 men (34.7%) and 98 women (65.3%) with a mean age of 54.93 ± 17.15 (years). The majority of participants had BMI (kg/m^2^) 27.08 ± 5.97. Most of them lived in urban areas (90%), 42% were illiterate, 63.3% were married, 93.3% lived with their families, and 46% were unemployed. A low percentage had medical coverage (28.7%) and 56.7% of the patients had a low annually family income < 36,000 (Dhs). The interviewed participants (T1D and T2D) had suffered from diabetes for an average of 11.29 ± 8.73 years with a dominance of T2D for 53.3% (Table 1).

**Table 1:** Socio-demographic and disease characteristics of the study participants

Variables	N (%)
Gender	
Male	52 (34.7%)
Female	98 (65.3%)
Place of residence	
Urban	135 (90%)
Rural	15 (10%)
Educational level	
Illiterate	63 (42%)
Primary/Koranic	25 (16.7%)
Middle School/High School	24 (16%)
University	38 (25.3%)
Marital status	
Single	31 (20.7%)
Married	95 (63.3%)
Divorced	5 (3.3%)
Widower	19 (12.7%)
Professional activity	
No occupation	69 (46%)
Student	9 (6%)
Employee	40 (26.7%)
Self-employed	9 (6%)
Retirement	23 (15.3%)
Insurance coverage	
No insurance	34 (22.7%)
Medical insurance	116 (77.3%)
Socioeconomic level (income (Dhs)/year)	
< 36,000	85 (56.7%)
36,000–72,000	45 (30%)
> 72,000	20 (13.3%)
Type of diabetes	
Insulin dependent (T1D)	70 (46.7%)
Not insulin dependent (T2D)	80 (53.3%)
Treatment	
Oral antidiabetic agents (T2D)	80 (53.3%)
Insulin (T1D)	51 (72.85%)
Oral antidiabetic agents per day + Insulin (T1D)	19 (27.15%)
Type of insulin (effect)	
Slow/semi-slow	42 (28%)
Fast	8 (5.3%)
Both	20 (13.3%)
Insulin doses per day	
Once/day	10 (6.7%)
2 times/day	48 (32%)
3 times/day	12 (8%)
Oral antidiabetic agents doses per day	
Once/day	26 (17.3%)
2 times/day	37 (24.7%)
3 times/day	36 (24%)
Comorbidities	
Yes	81 (54%)
No	69 (46%)
Type of comorbidity	
None	69 (46%)
Hypertension	48 (32%)
Renal failure	3 (2%)
Obesity/overweight	11 (7.3%)
Others	18 (12%)
Complications	
Yes	69 (46%)
No	81 (54%)
Housing	
Alone	10 (6.7%)
With family	140 (93.3%)
Adherence to dietary guidelines (pre-lockdown)	
Yes	113 (75.3%)
No	37 (24.7%)
Adherence to dietary guidelines (post-lockdown)	
Yes	106 (70.7%)
No	44 (29.3%)
Physical activity (≥ three times 30 min per week)	
Yes	92 (61.3%)
No	58 (38.7%)
Type of physical activity	
Walking	54.70%
Soccer	2.70%
Other (aerobics, cycling, taekwondo, running, etc.)	42.60%
Respect of appointments (pre-lockdown)	
Yes	116 (77.3%)
No	34 (22.7%)
Respect of appointments (during lockdown)	
Yes	87 (58%)
No	63 (42%)
Stopping medication (pre-lockdown)	
Yes	22 (14.7%)
No	128 (85.3%)
Stopping medication (during lockdown)	
Yes	31 (20.7%)
No	118 (78.7%)
Self-monitoring (pre-lockdown)	
Rarely or never	49 (32.7%)
If symptom	29 (19.3%)
2 times/week	11 (7.4%)
Once/day	61 (40.7%)
Self-monitoring (during lockdown)	
Rarely or never	55 (36.7%)
If symptom	23 (15.3%)
2 times/week	14 (9.3%)
Once/day	58 (38.7%)
Fasting (during lockdown)	
Yes	96 (64%)
No	54 (36%)
Anxiety about getting COVID-19 (during lockdown)	
Yes	91 (60.7%)
No	59 (39.3%)
COVID-19 infection	
Yes	15 (10%)
No	135 (90%)
COVID-19 vaccination	
Yes	90 (60%)
No	60 (40%)
Doses of COVID-19 vaccination	
1 dose	17 (18.9%)
2 doses	73 (81.1%)

### Primary outcome

Surprisingly, we found a significant improvement in post-lockdown mean ± SD HbA1c: 7.97 ± 1.57 versus 8.35 ± 1.97, *p* = 0.005. Hence, COVID-19 lockdown had no deleterious effect on HbA1c levels in people with T1D and T2D (Table 2).

**Table 2:** HbA1c mean ± SD before and after confinement

HbA1c	Mean ± SD	*p* value
Pre-lockdown	8.35 ± 1.97	
Post-lockdown	7.97 ± 1.58	0.005

### Secondary outcomes

No significant association was detected between HbA1c deviation and age, gender, weight, height, current BMI status, fasting blood sugar, family history, urban or rural areas, marital status, professional activity, socioeconomic income, type of diabetes, dietary, comorbidities, diabetic complications and housing.

No effect of lockdown on weight (kg) was observed: 72.43 ± 14.83 (pre-lockdown) versus 72.24 ± 14.49 (post-lockdown). On the other hand, we observed a slight decrease in adherence to dietary guidelines from 75.3% (pre-lockdown) to 70.7% (post-lockdown). The majority respected the dietary recommendations during the lockdown period. According to the recommendations of the Francophone Diabetes Society, (minimum level ≥ three times 30 min per week) (Duclos et al. 2013), we also reported a slight drop in the physical activity score (scale from 0 to 10): 5.06 ± 2.41versus 4.19 ± 2.48. Stay-at-home orders have had a negative impact on medical appointments. Only 58% of participants were able to consult their clinician during the confinement compared to the pre-lockdown period (77.3%). Eighty of 150 participants (53.3%) received oral antidiabetic agents, 34% were treated with insulin alone, and 12.7% received insulin and oral antidiabetic drugs. During the lockdown, 20.7% stopped the medication compared to 14.7% in pre-lockdown.An unremarkable change was observed in self-monitoring which increased by 4% (32.7% vs 36.7%) in patients who seldom or never self-monitor. During the lockdown period, 64% of participants declared fasting the holy month of Ramadan. Our study showed that 60.7% were anxious about getting COVID-19. In contrast, only 10% were affected by COVID-19 during confinement (Tables 1 and 3).

**Table 3:** Independent variables variation before and after lockdown

Variables	Mean ± SD	Median [Q1; Q3]
Age (years)	54.93 ± 17.15	58.00 [46; 67]
Height (cm)	163.8 ± 8.70	163.00 [158; 169]
Current BMI (kg/m^2^)	27.08 ± 5.97	26.00 [23; 29.72]
Diabetes duration (years)	11.29 ± 8.73	9.50 [4; 16]
Weight (pre-lockdown) (kg)	72.43 ± 14.83	70.00 [62; 82]
Weight (post-lockdown) (kg)	72.24 ± 14.49	70.00 [63; 80]
HbA1c (pre-lockdown) (%)	8.35 ± 1.97	8.00 [7; 10]
HbA1c (post-lockdown) (%)	7.97 ± 1.57	7.50 [7; 9]
Blood sugar (pre-lockdown) (g/l)	1.60 ± 0.57	1.50 [1.2; 2]
Blood sugar (post-lockdown) (g/l)	1.63 ± 0;50	1.54 [1.21; 2]
Physical activity score (pre-lockdown) (0 to 10)	5.06 ± 2.41	5.00 [3; 7]
Physical activity score (post-lockdown)) (0 to 10)	4.19 ± 2.48	5.00 [2; 6]

*BMI* body mass index, *HbA1c* glycated hemoglobin, *SD* standard deviation, *Q* quartile

No significant association was detected between HbA1c deviation and adherence to the dietary recommendations, physical activity, medical appointments, stopping medication, self-monitoring, fasting, and anxiety about getting COVID-19. However, a significant association was observed of HbA1c deviation with educational level and medical coverage (*p* = 0.01).

## Discussion

In our original study, we aimed to assess the COVID-19 lockdown impact on HbA1c levels in Moroccan patients with T1D and T2D. We detected an overall improvement in HbA1c value by the end of lockdown time compared to pre-lockdown period. The pre-lockdown mean ± SD HbA1c was 8.35% ± 1.97 versus 7.97% ± 1.57 after the lockdown. The HbA1c deviation was independent of the age, gender, weight, height, current BMI status, fasting blood sugar, family history, urban or rural areas, marital status, professional activity, socioeconomic income, type of diabetes, dietary, comorbidities, diabetic complications, housing, adherence to the dietary recommendations, physical activity, medical appointments, stopping medication, self-monitoring, fasting and anxiety about getting COVID-19. Our findings are consistent with several studies that demonstrated no effect of the COVID-19 lockdown on HbA1c. A study carried out by Anjana et al. in 2020 revealed an improvement in HbA1c (*n* = 205 T2D): post-lockdown HbA1c = 7.7 ± 1.7 versus 8.2 ± 1.9%, *p* < 0.001 (Anjana et al. 2020). A meta-analysis performed by Psoma et al. (2020) showed a decrease in HbA1c (%) (*n* = 380 T2D): pre-lockdown HbA1c (%) 6.9 ± 1.3 versus post-lockdown HbA1c (%) 6.7 ± 0.9; *p* = 0.015 (Psoma et al. 2020). A pre- and post-lockdown data from 110 adults with T2D noted no significant change in mean HbA1c (Sankar et al. 2020). Retrospective analysis conducted by Pla et al. (2020) detected an improvement in glycemic control in patients with T1D during COVID-19 lockdown (HbA1c: 7.21 ± 0.78% vs 6.83 ± 0.71%, *p* = 0.0005). Fernández et al. (2020) detected, after the lockdown, that the mean glucose declined from 166.89 ± 29.4 to 158.0 ± 29.0 mg/dL in patients with T1D (*n* = 307), and their HbA1c decreased from 7.4 ± 1.0 to 7.1 ± 1.0% (54 ± 10.9 vs 57 ± 10.9 mmol/mol; *p* < 0.001). In contrary, other studies described a deleterious effect of lockdowns on glycemic control. Recently, Khare and Jindal found a 0.51% increase in mean HbA1c values after home confinement (Khare and Jindal 2021). A study of 101 patients with T2D reported an increase in HbA1c value (from 7.67 ± 1.76 to 8.11 ± 2.48) (Önmez et al. 2020). The reported discoveries, although similar to our present study, demonstrated that the COVID-19 lockdown, regardless of the other risk factors, had no effect on diabetic state and related factors.

Possible reasons cited for the decrease in HbA1c levels in our study during lockdown were a more regular lifestyle: stability at home, spending more time with family, less stress due to teleworking conditions, less exposure to pollution, more time to prepare healthy meals, meals on time, increased contribution to household chores, better sleep and awareness of more serious risks complications of COVID-19 and comorbidities.

Difficulties in accessing exercise centers or outdoor exercise could explain the slight decrease in physical activity offset by household chores. The majority of participants maintained self-monitoring and compliance with dietary recommendations during the confinement period, which compensated for the difficulties in traveling to health centers for medical appointments.

Our finding reported a significant association in HbA1c deviation according to educational level and medical coverage. Moderate and high level of education could improve the glycemic control. Several studies have recorded the negative impact of low educational level on diabetic state (Larsson et al. 1999; Kim et al. 2017; Fiseha et al. 2018). The majority of patients have medical insurance, which facilitates access to care and ensures good patient care. Medical coverage can contribute significantly to improving glycemic levels. The cross-sectional study conducted by Canedo et al. 2018 explained the negative impact of the lack of insurance coverage on the quality of diabetes control (Canedo et al. 2018). Glantz et al. (2020) determined that health insurance influences the glycemic control (Glantz et al. 2020). These findings are consistent with our results; the lack of medical coverage constitutes a risk factor for diabetes management. Despite the application of strict confinement and the interruption of health care, patients with T1D and T2D did not experience a worsening of glycemic control, but rather an improvement.

## Conclusion

To conclude, the confinement due to COVID-19 did not cause a main disturbance in glycemic balance. Short duration of home confinement might be sufficient to affect significantly the glycemic control and other risk factors. Further work with larger study samples is needed to identify the metabolic changes that occurred after the strict lock-in period. Nonetheless, the slight improvement recorded implies an improvement in public health policies aiming at encouraging telemedicine and raising awareness about maintaining a healthy lifestyle.

## Data Availability

Not applicable.

## Abbreviations

COVID-19: Coronavirus disease of 2019
SARS-CoV-2: Severe acute respiratory syndrome coronavirus 2
T1D: Type 1 diabetes
T2D: Type 2 diabetes
WHO: World Health Organization
HbA1c: Glycated hemoglobin
SD: Standard deviation
BMI: Body mass index
OR: Odds ratio
